# Field evaluation of a topical combination of ivermectin, imidacloprid, and praziquantel for flea control in cats under routine clinical

**DOI:** 10.3389/fpara.2026.1755999

**Published:** 2026-02-25

**Authors:** Camilo Romero-Núñez, Ariadna Flores-Ortega, Rafael Heredia-Cárdenas, Enrique Salazar-Grosskelwing

**Affiliations:** 1 DERMAVET, Veterinary Hospital, México City, Mexico; 2 Centro Universitario Universidad Autónoma del Estado de México (UAEM) Amecameca, Amecameca, Estado de México, Mexico; 3 CIVET, Veterinary Integral Center, Animal Health and Welfare, Ecatepec, Mexico, Mexico; 4 Laboratory of Parasitology, Diagnostic Unit “Rancho Torreón del Molino”, Faculty of Veterinary Medicine and Zootechnics, Universidad Veracruzana, Veracruz, Mexico

**Keywords:** companion animals, *Ctenocephalides felis*, ectoparasite control, feline dermatology, field study, flea infestation, imidacloprid, ivermectin

## Abstract

**Introduction:**

Flea infestation remains a major clinical and public health concern in cats due to its negative impact on animal welfare and its role in the transmission of zoonotic pathogens. Effective flea control under routine field conditions is essential for integrated parasite management in companion animals. The aim of this study was to evaluate the clinical performance of a topical formulation combining ivermectin, imidacloprid, and praziquantel for flea control in domestic cats under field conditions.

**Methods:**

A total of 142 naturally infested cats of varying ages, sexes, and body conditions were included in the study. All animals received a single topical application of the evaluated formulation. Clinical and parasitological assessments were performed on Days 0, 1, 7, 14, and 30 post-treatment using standardized flea counts, pruritus scores, and dermatological lesion scores. Treatment effectiveness was assessed using within-subject pre–post comparisons. Effective flea control was defined as a marked reduction in the proportion of flea-positive cats relative to baseline, accompanied by concurrent clinical improvement.

**Results:**

The proportion of cats free of detectable fleas exceeded 91% by Day 7 and approached 95% by Day 30 post-treatment. Significant clinical improvement was observed during the first two weeks, including reductions in pruritus intensity and dermatological lesion scores. Treatment response did not differ according to sex or age, indicating consistent clinical performance across diverse feline subpopulations. No adverse effects were reported throughout the study period.

**Discussion:**

The findings indicate that the evaluated topical combination provides consistent and clinically meaningful flea control in domestic cats under routine field conditions, supporting its use as part of integrated parasite management strategies in companion animals. However, the absence of a negative control or active comparator group represents a limitation of the study; therefore, treatment effectiveness was assessed using within-subject comparisons rather than established regulatory efficacy benchmarks.

## 1 Introduction

Cats live in close contact with humans, with many even sleeping in the same bed as their owners. It is also common for cats to have access to outdoor areas where they face a high likelihood of exposure to ectoparasites and pathogens (Razgūnaitė et al., 2021). Fleas are ectoparasites of major medical and veterinary importance; they are frequently encountered in companion-animal practice, infest households, and represent a significant nuisance for pet owners. Flea-associated diseases account for approximately half of the dermatological cases presented to small-animal veterinarians. Their bites typically cause irritation and allergic reactions, and fleas are capable of transmitting numerous zoonotic pathogens, including the cestode *Dipylidium caninum*, filarial endoparasites, as well as protozoa and bacteria such as *Yersinia pestis, Francisella tularensis, Bartonella, Ehrlichia, Rickettsia*, and *Mycoplasma*. *Babesia microti* and *Toxoplasma gondii* have also been detected in fleas; however, additional studies are required to confirm their ability to transmit these agents (Farrell et al., 2022; Lavan et al., 2021; Pawełczyk et al., 2020).

Fleas have developed multiple modes of pathogen transmission, including blood feeding, infected feces, and vertical, horizontal, and mechanical routes (Pawełczyk et al., 2020). Ctenocephalides felis easily completes its life cycle by feeding on pets within households or in peridomestic environments where temperature and humidity conditions are suitable for sustaining immature flea stages. This species does not appear to be strongly host-specific; it has been suggested that flea populations can be maintained in wildlife, which may explain their persistence in domestic infestations (Farrell et al., 2022).

Given the high frequency of close contact between cats and their owners, as well as between cats and other domestic animals, cats may play an important role in maintaining and transmitting zoonotic agents to humans (Razgūnaitė et al., 2021). The introduction of monthly topical veterinary ectoparasiticides has markedly improved flea control, allowing effective management of infestations through a single treatment application and enhancing user convenience (Lavan et al., 2021).

Imidacloprid is a well-established neonicotinoid insecticide widely used as a topical flea adulticide in cats, acting as an agonist of insect nicotinic acetylcholine receptors and producing rapid knockdown and sustained control of *Ctenocephalides felis* under field conditions (Marchiondo et al., 2007; Lavan et al., 2021). However, cats presented for veterinary care frequently harbor concurrent parasitic infections that are not addressed by imidacloprid alone. Ivermectin, a macrocyclic lactone, exerts its antiparasitic activity through modulation of glutamate-gated chloride channels and is effective against a broad range of nematodes and arthropods; although it is not primarily classified as a flea adulticide, macrocyclic lactones are commonly incorporated into combination products to expand parasite coverage and support integrated parasite management strategies (McTier et al., 2000; Zakson-Aiken et al., 2001). Praziquantel is included for its established cestocidal efficacy, particularly against *Dipylidium caninum*, a flea-transmitted zoonotic tapeworm frequently diagnosed in cats with flea infestations (Bowman et al., 2002; ESCCAP, 2021). Therefore, the rationale for evaluating this combination is not to enhance the known flea adulticidal activity of imidacloprid, but to assess the clinical performance of a broad-spectrum formulation designed to simultaneously address flea infestation and flea-associated parasitic risks under real-world conditions (Cermolacce et al., 2023). Therefore, the aim of this study was to evaluate the effectiveness of a topical combination of ivermectin, imidacloprid, and praziquantel against fleas in cats under field conditions.

## 2 Methodology

The study was conducted in Mexico City, Mexico, under field (clinical) conditions. Mexico City is a high-altitude urban area (~2,240 m above sea level) characterized by a temperate subhumid climate with marked seasonal variation, including a rainy season typically extending from late spring to early autumn. Mean annual temperatures are moderate, while indoor microclimates in urban households often provide stable temperature and humidity conditions that favor the development of flea life stages throughout the year. High human and pet population density, close human–animal cohabitation, and predominantly indoor or semi-indoor pet management practices may further influence flea exposure and reinfestation dynamics in this setting. All 142 cats enrolled in the study were managed in accordance with ISFM/AAFP guidelines (Rodan et al., 2022).

### 2.1 Animals

A total of 142 cats were enrolled in the study using a consecutive convenience sampling approach. All cats presented to the participating veterinary clinics during the study period were screened consecutively for eligibility, and those fulfilling the predefined inclusion criteria were enrolled until the target sample size was achieved. This recruitment strategy was selected to reflect routine clinical practice and to capture a heterogeneous population of naturally flea-infested cats under field conditions. The study population included 69 females and 73 males, comprising 49 kittens, 39 juveniles, 45 adults, and 9 senior cats. Body condition scores were classified as low (19 cats), ideal (103 cats), and high (20 cats). Ninety-six cats were gonadectomized and 46 were intact. This investigation was designed as a field-effectiveness evaluation under routine clinical conditions, focusing on cats naturally infested with fleas and presented for veterinary care. For ethical and animal welfare reasons, the inclusion of an untreated negative control group was not considered acceptable, as withholding antiparasitic treatment from cats with active flea infestation could result in sustained pruritus, dermatological lesions, discomfort, and an increased risk of pathogen transmission. Similarly, the use of an active comparator group (e.g., imidacloprid alone or another registered flea control product) would have required randomization and blinding procedures that were not compatible with the real-world clinical setting or with the primary objective of this observational study. Consequently, each animal served as its own control, and treatment response was assessed by comparing post-treatment parasitological and clinical outcomes with baseline values obtained prior to treatment administration.

Written informed consent was obtained from all owners before enrollment. To be eligible for inclusion, cats were required to be naturally infested with at least five fleas, and neither the animals nor their environment could have received any ectoparasitic treatment during the four weeks preceding enrollment. Environmental flea control measures were not standardized or actively monitored during the study period, as the investigation was designed to reflect routine clinical conditions. Owners were instructed not to apply additional ectoparasitic treatments to the animals or to the household environment during follow-up; however, environmental exposure to flea reservoirs, including immature stages present in household or peridomestic environments, could not be fully controlled. Accordingly, the study primarily evaluated the performance of the topical treatment under real-world conditions rather than under controlled environmental settings.

### 2.2 Parasitological and clinical evaluations

Day 0 was defined individually as the day on which each animal was assessed and confirmed as eligible for enrollment. On Day 0, all required parasitological and clinical examinations were performed. Flea counts were conducted following a standardized protocol (Marchiondo et al., 2007). Each cat was combed with a fine-toothed flea comb over the entire body for a total of 7 minutes, including the dorsal midline (2 min), tail head (2 min), left side (1 min), right side (1 min), and inguinal region (1 min). After fleas were counted, they were returned to the animal to preserve the natural infestation. The same assessments were repeated on Days 1, 7, 14, and 30. All flea counts and parasitological assessments were performed by the same trained veterinarian throughout the study to minimize inter-observer variability, and the standardized flea-combing protocol was consistently applied at all evaluation time points. Blinding of the evaluator was not feasible due to the field-effectiveness study design and the absence of a control or comparator group; however, the use of a single evaluator and a predefined counting protocol was intended to reduce potential measurement bias. Each cat underwent a clinical examination at each visit, and dermatological lesions were recorded using the validated Scoring Feline Allergic Dermatitis (SCORFAD) scale (Steffan et al., 2012). In all cases, a pruritus visual analog scale adapted for cats (0–10) was used (Noli et al., 2019). A questionnaire was administered to collect additional epidemiological data. All evaluations were performed in the clinic. The questionnaire was designed as a structured epidemiological form to collect complementary background information rather than to generate outcome variables or perform inferential analyses. It included standardized items addressing basic signalment confirmation, housing conditions (indoor, outdoor, or mixed), contact with other animals, recent travel history, and prior ectoparasite control practices. The questionnaire did not influence eligibility criteria, treatment allocation, or outcome assessment, and its data were used descriptively to contextualize flea exposure under routine clinical conditions.

Adverse events were monitored using a combined approach consisting of (i) active veterinary clinical assessment at each scheduled evaluation (Days 0, 1, 7, 14, and 30) and (ii) structured owner-reported follow-up. At each visit, cats underwent a brief physical examination and targeted neurologic observation (mentation, gait/ataxia, tremors, pupil size, hypersalivation). Owners were asked standardized questions using a checklist addressing potential post-treatment events, including hypersalivation, vomiting, diarrhea, lethargy, anorexia, exacerbation of pruritus, erythema at the application site, ataxia, tremors, and mydriasis. Any suspected adverse event was recorded with respect to onset time, duration, severity, and outcome. Owners were instructed to contact the clinic immediately if abnormal clinical signs occurred between scheduled visits. Flea control effectiveness was determined by comparing the presence of live fleas before treatment with findings obtained during post-treatment evaluations. No medications that could influence parasitological or clinical assessments were permitted during the study period.

### 2.3 Treatment

After parasitological and clinical evaluations were completed, each cat received treatment with Feline Full Spot^®^ (Imidacloprid 11 g, Ivermectin 0.2 g, and Praziquantel 7.5 g/100ml). A single dose was applied directly onto the skin at three points along the dorsal midline, from the nape of the neck to the withers, to minimize the risk of licking. No patient received any additional topical or systemic treatment during the evaluation period.

### 2.4 Statistical analysis

A Shapiro–Wilk normality test was performed to assess data distribution. Because the data were not normally distributed, non-parametric tests were used. A matched-pairs test was applied to compare pruritus scores and lesion scores across time points. For epidemiological variables, a chi-square test was used to evaluate associations between host-related variables and flea presence. In addition to hypothesis testing, descriptive effect estimates were calculated to support the interpretation of results. Proportions of flea-positive and flea-free cats at each evaluation time point were accompanied by 95% confidence intervals (CI) calculated using exact binomial methods. Because the study was designed as an exploratory field-effectiveness evaluation with a limited number of predefined within-subject comparisons, no formal correction for multiple comparisons was applied; results were interpreted with emphasis on effect magnitude and consistency across time points rather than on isolated p-values. All analyses were conducted using JMP statistical software version 8.0.

## 3 Results

All cats (n = 142) were infested with fleas at baseline (Day 0). Treatment effectiveness was assessed using within-subject comparisons between baseline and post-treatment evaluations. In this study, effective flea control was defined as a reduction in the proportion of cats with detectable live fleas relative to Day 0, together with concurrent improvement in clinical parameters. Flea infestation was analyzed as a binary outcome (presence or absence of live fleas) rather than as mean flea counts. This approach was selected to reflect a clinically relevant endpoint in routine veterinary practice, where flea-free status and resolution of clinical signs are primary indicators of treatment success. Additionally, binary outcomes reduce variability associated with flea counting techniques under field conditions and facilitate interpretation of effectiveness in heterogeneous patient populations. Using these criteria, the proportion of flea-positive cats decreased from 100% at baseline to 8.39% (11/142) by Day 7, corresponding to a reduction of 91.61%. On Days 14 and 30, fleas were detected in 8 (5.97%) and 7 (5.18%) cats, representing reductions of 94.03% and 94.82%, respectively (**Table 1**).

**Table 1:** Proportion and percentage of cats with fleas following treatment with FELINE full spot^®^.

Cats (n = 142)	Day 1	Day 7	Day 14	Day 30
Presence/absence of fleas	142/0	11/131	8/134	7/135
% presence of fleas	100	8.39	5.97	5.18
% reduction vs. baseline	0	91.61	94.03	94.82

a Reduction percentages were calculated relative to baseline (Day 0).

b Flea infestation was analyzed as a binary outcome (presence/absence of live fleas).

c Flea-positive proportions are reported with 95% confidence intervals (exact binomial) in the Results section.

The proportion of flea-positive cats at each time point was further characterized by 95% confidence intervals to quantify the precision of the estimates. At Day 7, the flea-positive proportion was 8.39% (11/142; 95% CI: 4.25–14.50%), decreasing to 5.97% at Day 14 (8/142; 95% CI: 2.60–11.39%) and to 5.18% at Day 30 (7/142; 95% CI: 2.10–10.41%). These intervals indicate a consistent and sustained reduction in flea infestation relative to baseline.

**Table 2** summarizes the changes in pruritus and dermatological lesion scores over time. Pruritus scores decreased markedly from Day 1 to Day 7 (5.30 to 0.33; p < 0.0001) and from Day 7 to Day 14 (0.33 to 0.24; p = 0.02), with statistically significant differences observed in both intervals. No significant difference was detected between Days 14 and 30. Similarly, lesion scores showed a substantial reduction from Day 1 to Day 7 (72.70 to 5.59; p < 0.0001) and from Day 7 to Day 14 (5.59 to 1.64; p = 0.004), while no statistically significant change was observed between Days 14 and 30.

**Table 2:** Comparison of mean pruritus scores and lesion scores in cats with flea infestation treated with FELINE full spot^®^.

Variable	Day 1–7	Day 7–14	Day 14–30
Pruritus (mean score)	5.30 → 0.33	0.33 → 0.24	0.24 → 0.23
P-value	< 0.0001*	0.02*	0.42
Lesions (mean score)	72.70 → 5.59	5.59 → 1.64	1.64 → 1.57
P-value	< 0.0001*	0.004*	0.43

*Matched-pairs test, 95% confidence level; significant.

**Table 3** presents the results of chi-square analyses evaluating associations between flea presence and host-related variables. No statistically significant associations were detected between flea presence and sex or age at any evaluation time point, indicating that treatment effectiveness was consistent across the evaluated feline subpopulations.

**Table 3:** General characteristics of the cats and their association with flea presence during 133 evaluations.

Evaluation day	Variable	χ²	P-value	OR	95% CI
Day 7	Sex	0.047	0.82	1.14	0.33–3.94
Day 7	Age	2.83	0.41	—	—
Day 14	Sex	0.007	0.93	0.86	0.63–1.19
Day 14	Age	3.27	0.35	—	—
Day 30	Sex	0.21	0.64	0.86	0.63–1.19
Day 30	Age	2.04	0.56	—	—

*Chi-square test; OR = odds ratio; 95% CI = 95% confidence interval; significant

No statistically significant associations were detected between flea presence and sex or age at any evaluation time point.

## 4 Discussion

The findings of this study demonstrate that the topical combination of imidacloprid, ivermectin, and praziquantel (Feline Full Spot^®^) provides consistent and clinically meaningful control of *Ctenocephalides felis* in domestic cats under field conditions. Using predefined evaluation criteria based on within-subject comparisons, a marked reduction in flea infestation was observed, with a 91.61% decrease in flea-positive animals by Day 7 and a sustained reduction reaching 94.82% by Day 30. These results are consistent with previous reports describing the clinical effectiveness of long-acting topical ectoparasiticides, including studies documenting high levels of effectiveness and owner satisfaction with transdermal flea control formulations in cats (Lavan et al., 2021) and in other species under comparable field conditions (Cermolacce et al., 2023).

In parallel with the parasitological response, a statistically significant improvement in clinical parameters was observed during the first two weeks following treatment. Reductions in pruritus intensity and dermatological lesion scores indicate not only successful flea control but also an improvement in animal welfare, which is a central objective in contemporary feline medicine (Rodan et al., 2022). The magnitude and temporal pattern of clinical improvement observed in this study are comparable to outcomes reported in previous investigations of flea control products evaluated under similar conditions (Farrell et al., 2022). Treatment response did not differ significantly according to sex, age, or body condition, suggesting consistent clinical performance across diverse feline subpopulations. This finding supports the practical applicability of the formulation in routine veterinary practice, where patient populations are inherently heterogeneous. Although certain studies have identified age or seasonal factors as modifiers of flea infestation risk (Farrell et al., 2022), such variability may be more strongly influenced by environmental and geographic conditions than by host-related factors alone.

The findings of this study should be interpreted in light of the climatic, ecological, and management characteristics specific to Mexico City. Flea development and survival are strongly influenced by temperature, humidity, and the availability of suitable microhabitats, particularly indoor environments that may buffer seasonal climatic extremes (Dryden and Rust, 1994; Rust and Dryden, 1997). Urban housing conditions, high-density pet populations, and frequent indoor or semi-indoor confinement of cats may contribute to sustained flea exposure and reinfestation pressure, potentially affecting observed treatment performance. Consequently, while the results demonstrate consistent and clinically meaningful flea control under the field conditions evaluated, extrapolation to other geographic regions such as areas with different climates, rural environments, or alternative pet management practices should be made with caution. Additional studies conducted across diverse ecological and climatic settings would be valuable to further define regional variability in treatment effectiveness.

Despite the overall level of effectiveness observed, a small proportion of cats exhibited persistent or possibly new flea infestations by Day 30. The presence of fleas in this subset of animals should be interpreted in the context of uncontrolled environmental exposure. Flea life stages developing in household and peridomestic environments are a well-recognized source of reinfestation, particularly when environmental treatments are not concurrently implemented. Because environmental control measures were not standardized or actively monitored in this study, residual flea presence may reflect ongoing exposure to environmental reservoirs rather than reduced product effectiveness. These findings underscore the importance of integrated flea management strategies that combine on-animal treatment with environmental interventions to achieve sustained control, especially in endemic settings (Pawełczyk et al., 2020; Cermolacce et al., 2023).

Although the formulation evaluated combines imidacloprid, ivermectin, and praziquantel, the present study design does not allow differentiation of the individual contributions of each active ingredient to the observed flea control. Imidacloprid is recognized as the primary flea adulticide within the formulation, and its efficacy as a standalone compound against *C. felis* in cats has been extensively demonstrated in controlled laboratory and field studies (Marchiondo et al., 2007; Lavan et al., 2021). Macrocyclic lactones such as ivermectin contribute to broad-spectrum parasite control and are widely used in combination endectoparasiticides; however, their direct contribution to flea adulticidal efficacy is considered limited compared with neonicotinoids (McTier et al., 2000; Zakson-Aiken et al., 2001). Praziquantel targets cestode infections such as *Dipylidium caninum*, which are epidemiologically linked to flea infestation, but it does not exert direct insecticidal effects (Bowman et al., 2002). Consequently, the observed effectiveness reported here reflects the clinical performance of the combination as a whole under real-world conditions rather than additive or synergistic flea-specific effects attributable to all components.

The effectiveness observed in this study should therefore be interpreted within the context of the predefined evaluation criteria and study design. Terms such as “effective” or “rapid reduction” refer to the magnitude of change observed in flea presence and clinical scores when compared with baseline values within the same animals, rather than to established regulatory efficacy thresholds derived from controlled comparative trials. Accordingly, the results demonstrate a consistent and clinically meaningful reduction in flea infestation and associated clinical signs under routine clinical conditions, while avoiding direct inference regarding regulatory-level or comparative efficacy. Future randomized controlled studies incorporating active comparators would be necessary to further define relative efficacy and the individual contribution of each component of the formulation.

Although no adverse events were detected in the present study, safety considerations remain important when macrocyclic lactones such as ivermectin are used in cats. Reported adverse effects associated with ivermectin exposure are typically linked to overdose, inappropriate formulations, or accidental ingestion and primarily involve neurologic signs due to central nervous system penetration, including lethargy, ataxia, hypersalivation, mydriasis, tremors, seizures, and coma (Merola and Eubig, 2012). Feline cases of ivermectin toxicosis have been described in the literature, including severe presentations following substantial overdoses (Lewis, 1994; Muhammad et al., 2004). Therefore, appropriate product selection, correct dosing, and client counseling to prevent accidental ingestion or misuse remain essential. Under the conditions and dosing regimen applied in this field study, the formulation was well tolerated throughout the 30-day monitoring period, supporting its practical use in clinical settings in accordance with feline-friendly handling and treatment recommendations (Rodan et al., 2022).

## Data Availability

The original contributions presented in the study are included in the article/supplementary material. Further inquiries can be directed to the corresponding author.
